# Sensing Zn^2+^ in Aqueous Solution with a Fluorescent Scorpiand Macrocyclic Ligand Decorated with an Anthracene Bearing Tail

**DOI:** 10.3390/molecules25061355

**Published:** 2020-03-17

**Authors:** Matteo Savastano, Matteo Fiaschi, Giovanni Ferraro, Paola Gratteri, Palma Mariani, Antonio Bianchi, Carla Bazzicalupi

**Affiliations:** 1 Department of Chemistry “Ugo Schiff”, University of Florence, Via della Lastruccia, 3-13, 50019 Sesto Fiorentino, Italy; matteo.savastano@unifi.it (M.S.); mattefiaschi97@gmail.com (M.F.); giovanni.ferraro@unifi.it (G.F.); palma.mariani@unifi.it (P.M.); carla.bazzicalupi@unifi.it (C.B.); 2 Department of NEUROFARBA-Pharmaceutical and Nutraceutical section, and Laboratory of Molecular Modeling Cheminformatics & QSAR, University of Florence, Via Ugo Schiff 6, 50019 Sesto Fiorentino, Italy; paola.gratteri@unifi.it

**Keywords:** chemosensor, zinc, fluorescence, scorpiand, azamacrocycles, anion binding, supramolecular interactions

## Abstract

Synthesis of the new scorpiand ligand L composed of a [9]aneN_3_ macrocyclic ring bearing a CH_2_CH_2_NHCH_2_-anthracene tail is reported. L forms both cation (Zn^2+^) and anion (phosphate, benzoate) complexes. In addition, the zinc complexes of L bind these anions. The equilibrium constants for ligand protonation and complex formation were determined in 0.1 M NaCl aqueous solution at 298.1 ± 0.1 K by means of potentiometric (pH-metric) titrations. pH Controlled coordination/detachment of the ligand tail to Zn^2+^ switch on and off the fluorescence emission from the anthracene fluorophore. Accordingly, L is able to sense Zn^2+^ in the pH range 6–10 down to nM concentrations of the metal ion. L can efficiently sense Zn^2+^ even in the presence of large excess of coordinating anions, such as cyanide, sulphide, phosphate and benzoate, despite their ability to bind the metal ion.

## 1. Introduction

Scorpiand type aza-macrocyclic ligands, that is aza-macrocyclic ligands with coordinating tails [1], and their metal complexes have attracted considerable interest thanks to the numerous uses they have been tested for, including but not limited to various biomedical applications [2,3] (MRI contrast agents [4,5], radioisotopes complexation and radiolabelling [6,7,8,9], radiotherapy [10,11,12,13,14], chelation therapy [15,16,17], antiproliferative treatments [18,19,20,21], enzyme mimicking [22,23,24,25,26,27,28,29]), catalysis [30,31,32,33,34,35,36] and chemosensing [37,38,39,40,41,42,43]. 

As regards the latter, receptors that signal their interaction with substrate species through a modification of their fluorescence emission properties (fluorescent chemosensors) have aroused wide interest, in particular for their efficiency, in terms of sensitivity and response times, as well as for their relatively easy preparation and for their ability to detect a variety of chemical species [37,38,39,40,41,42,43,44,45,46,47,48,49,50].

Fluorescent chemosensors based on polyamines have proved very efficient for signalling both metal cations and anions in solution, even in water, where most chemosensors that function in less polar solvents fail to give adequate responses due to low solubility or weak binding to the target species. To this purpose, either chelation enhancement of fluorescence (CHEF) or chelation enhancement of quenching (CHEQ) experienced by the fluorophore upon interaction with the target species can be used. These fluorescent chemosensors are constituted by a binding site and a fluorophore communicating between them: the fluorophore can be either integrated into the binding site (intrinsic chemosensors) or appended to the latter through a spacer (conjugated chemosensors) [51]. 

**Figure 1:**
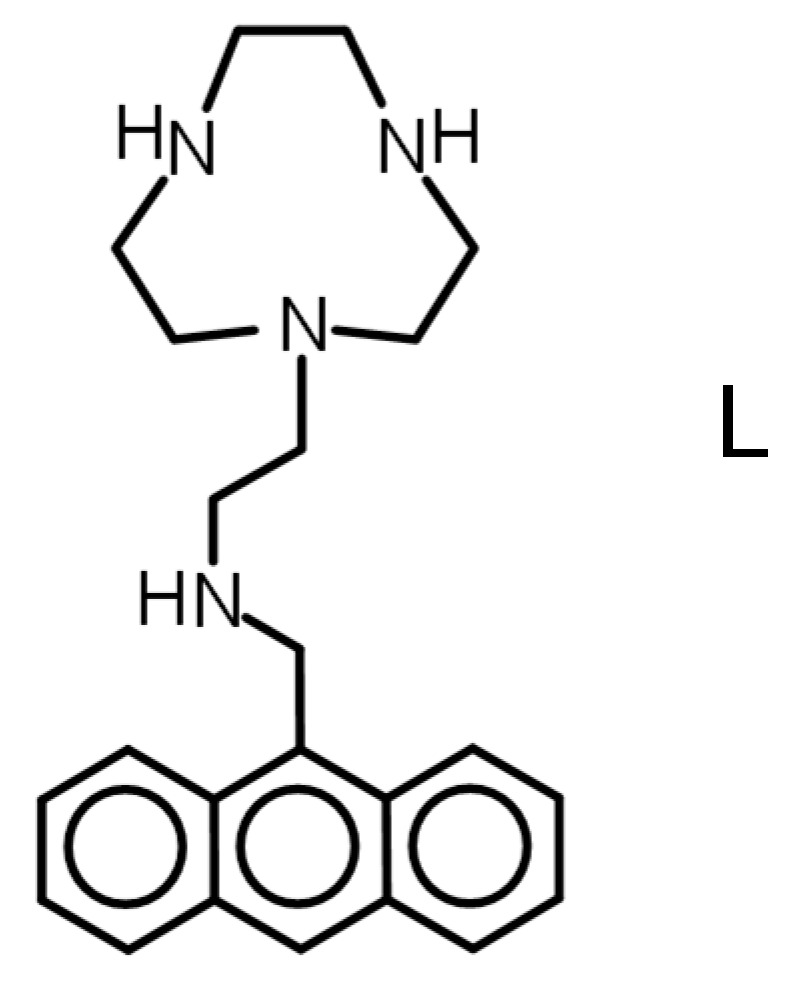
The ligand L.

Here, we describe the synthesis of the new conjugated chemosensor L (Figure 1) and its behaviour in sensing Zn(II) ions in water. L is a scorpiand type molecule constituted by a macrocyclic [9]aneN_3_ (1,4,7-triazacyclononane) binding unit connected to an anthracene fluorophore through a linker containing an additional nitrogen donor. [9]aneN_3_ has been widely used for the construction of chemosensors thanks to its peculiar coordination properties and the availability of consolidated synthetic procedures for its symmetric and non-symmetric functionalization [52].

In water, L is expected to bind either metal ions, when all or almost all amine groups are not protonated, or anions, when enough amine groups are protonated. In principle, interaction with both substrate types could affect the emission properties of the anthracene fluorophore.

Zn^2+^ was our first choice to test the new chemosensor as this metal ion plays an important role in biological systems [53] and commonly provides an efficient response to polyamine fluorescent chemosensors through the CHEF effect [51]. As L contains only four donor atoms, it is not able to saturate the coordination sphere of Zn^2+^, which could attract additional ligands present in solution, especially counter-anions, that might interfere with the sensing process. To address this possibility, sensing of Zn^2+^ with L was studied in the presence of coordinating anions such as phosphate (HPO_4_^2−^, H_2_PO_4_^−^), benzoate (Bz^−^), cyanide (CN^−^) and sulphide (S^2−^), showing that, even when they are in large excess, there is little to no interference.

## 2. Results and Discussion

### 2.1. Ligand Protonation

The equilibrium constants determined for the protonation of L in 0.1 M NaCl at 298.1 ± 0.1 K are reported in Table 1. 

**Figure 2:**
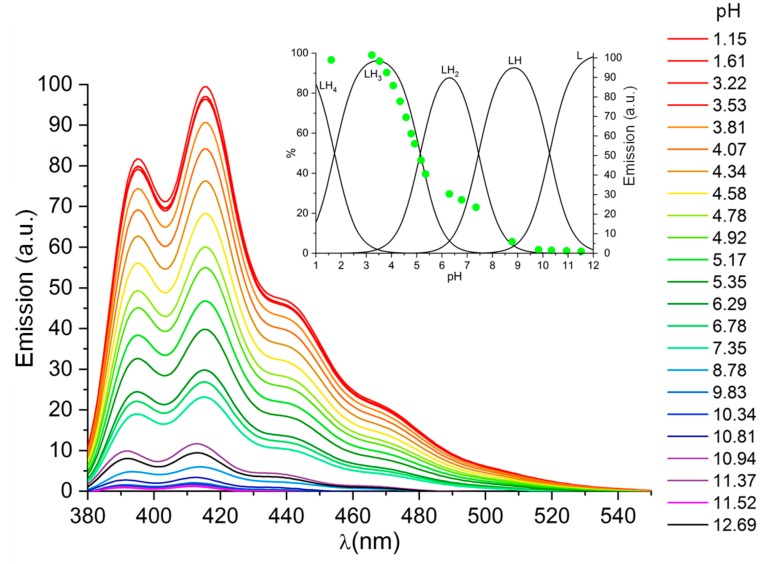
Emission spectra of ligand L at different pH values (λ_exc_ 362 nm). Inset: distribution diagrams of the species formed by L as a function of pH and emission intensities (dots) at 416 nm. [L] = 1 × 10^−5^ M.

**Figure 3:**
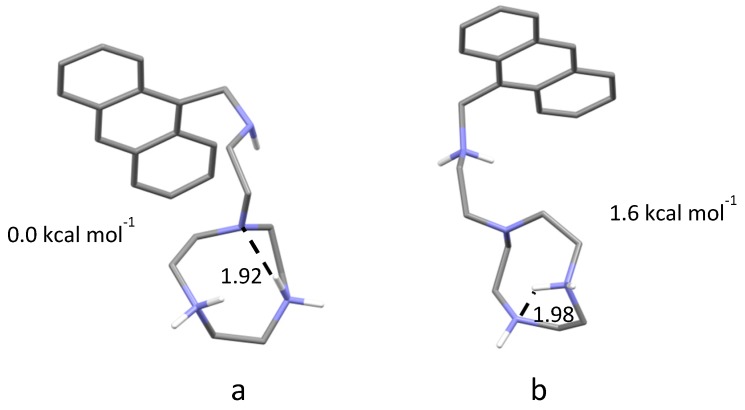
Calculated conformations for H_2_L^2+^; H-bonds shown as dashed lines (distances in Å). (**a**) Lower energy conformer. (**b**) Higher energy conformer. Energies calculated with respect to structure a.

As can be seen from this table, L behaves as tetraprotic base, in agreement with the presence of four amine groups in its structure. The equilibrium constants for the binding of successive protons decreases with increasing ligand protonation, according to the general behaviour of similar polyamines [54]. Based on previous data [54], the highest (logK = 10.26) and the lowest (logK = 1.74) constants can be assigned, respectively, to secondary and tertiary amine groups of the macrocyclic ring, while the assignment of the intermediate protonation constants required additional information. In this respect, the variation of the fluorescence emission properties of L with pH is greatly instructive. As shown in Figure 2, the emission spectra of L are characterized by the typical bands of anthracene. The molecule is strongly emissive in acidic solution, but its emission rapidly decreases above pH 3.5 to be completely quenched above pH 9.5. According to previous studies, quenching of the emission from similar molecules is expected to occur by photoinduced electron transfer (PET) to the excited anthracene fluorophore from the lone pair of a close amine group, such as the secondary amine groups of the ligand tail [51]. Protonation of this amine group prevents the PET process, activating the emission. The inset of Figure 2, where the emission at 416 nm (excitation at 362 nm) is superimposed to the distribution diagram of the species formed by L as a function of pH, clearly shows that the fluorescence emission of L starts being activated upon protonation of HL^+^ to form H_2_L^2+^ to reach its maximum with the formation of H_3_L^3+^, that is, the amine group of the ligand tail starts being involved in protonation, albeit partly, from the second protonation stage, becoming completely involved with the third one. Therefore, the partial emissive character of H_2_L^2+^ could be due to some sharing of H^+^ ions between the amine nitrogen of the ligand tail and the secondary nitrogen atoms of the macrocycle. To shed some light on this, we performed modelling calculations on the solvated H_2_L^2+^ species (see experimental section). Two conformers of similar energy were found. In the lowest energy one, both acidic hydrogen atoms are localized on the secondary nitrogen atoms of the macrocycle (Figure 3a), while in the other conformer the acidic hydrogen atoms are on a secondary amine group of the macrocycle and on the nitrogen atom of the ligand tail (Figure 3b). The calculated energy difference between these pair of conformers is small (1.6 kcal/mol) so that both conformers should be populated at room temperature, albeit with different abundance, in agreement with the fluorescence emission results. 

The absorption properties of L in the 320–400 nm range are substantially in agreement with this behaviour (Figure S1).

### 2.2. Zn^2+^ Complexation and Sensing

**Figure 4:**
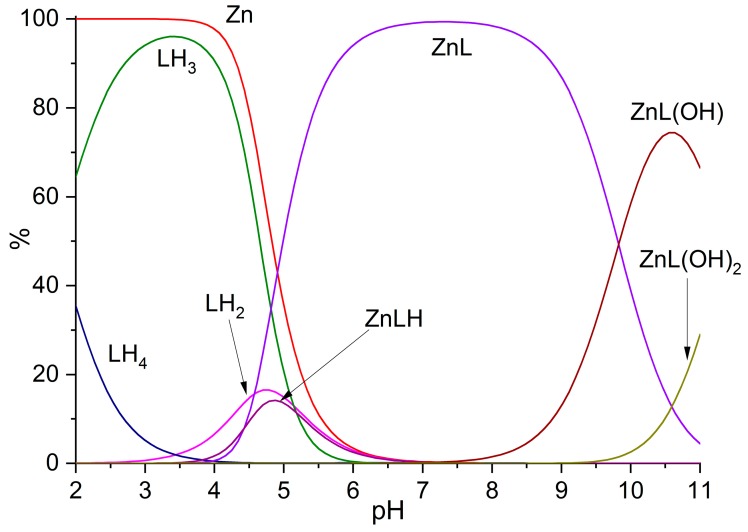
Distribution diagram of the species formed in the system Zn^2+^/L as a function of pH. [Zn^2+^] = [L] = 1 × 10^−3^ M. Charges omitted for simplicity.

Table 2 lists the equilibrium constants for the formation of Zn^2+^ complexes with L determined in 0.1 M NaCl at 298.1 ± 0.1 K. As shown by the distribution diagram of the species formed in solution as a function of pH (Figure 4), the ZnL^2+^ complex is largely prevalent in a wide pH range, being almost the unique species between pH 5.5 and 9. Protonation of ZnL^2+^ gives rise to the ZnHL^3+^, which is present in a very small proportion around pH 5, while the mono- and di-hydroxo complexes ZnL(OH)^+^ and ZnL(OH)_2_ are formed above pH 9 due to deprotonation of coordinated water molecules. 

**Figure 5:**
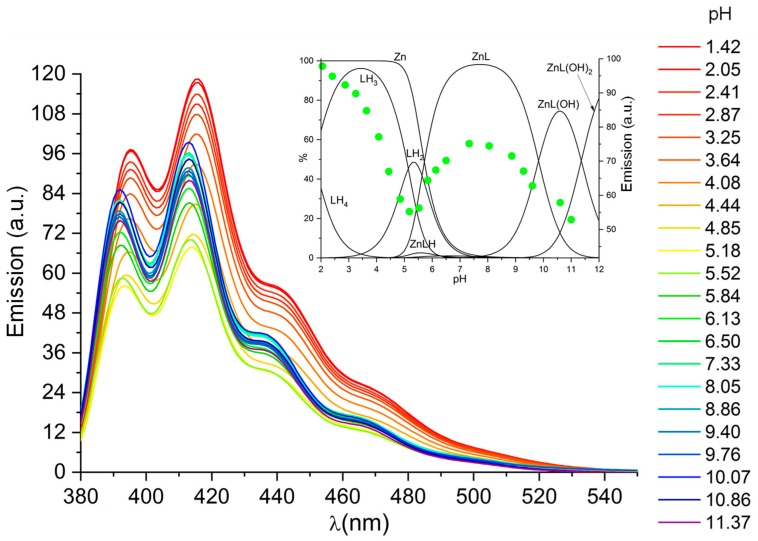
Emission spectra of Zn^2+^/L at different pH values (λ_exc_ 362 nm). Inset: distribution diagrams of the species formed as a function of pH and emission intensities (dots) at 416 nm. [L] = [Zn^2+^] = 1 × 10^−5^ M.

**Figure 6:**
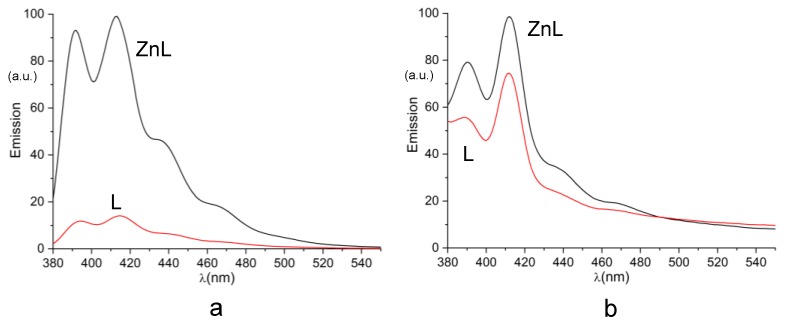
Emission spectra of L and Zn^2+^/L complexes, at pH 9, for: (**a**) [Zn^2+^] = [L] = 1 × 10^−6^ M, (**b**) [Zn^2+^] = [L] = 1 × 10^−9^ M. λ_exc_ 362 nm.

The fluorescence emission spectra recorded for the Zn^2+^/L system at different pH values (pH 1.42–11.37, Figure 5) show that, in acidic solutions, before complexation of Zn^2+^ starts taking place, the system shows the on-off emission profile observed in the absence of Zn^2+^ (Figure 2), while above pH 6 it returns to be emissive, in a pH range where otherwise it would be completely or almost completely quenched in the absence of this metal ion. For instance, the ratio between quantum yields of Zn^2+^/L and L at pH 9 is 11.1. Such revival of the emission upon complexation means that metal coordination of the amine groups of the ligand tail prevents quenching of the emission via the PET process. Nevertheless, as previously observed for similar systems [51], a significant emission decrease is observed in alkaline solutions, where hydroxo-complexes are formed. Such bell-shaped profile of the emission intensity versus pH defines the pH region (6–10) in which L can be used as fluorescent chemosensor for Zn^2+^ (Figure 5). For instance, in the range of μM concentrations, a nine-fold increase of emission is observed between L and ZnL^2+^ at pH 9. The emission difference decreases in more diluted solutions, but even in the nM concentration range is still appreciable (Figure 6). As a matter of fact, the limit of detection (LOD) for Zn^2+^, referred to the addition of the metal ion to a 1 × 10^−5^ M solution of L, was determined to be 30 nM based on the 3σ/slope of the fitting curve (Figure S2).

Interestingly, also absorption spectra recorded in the 320–400 nm range are able to signal Zn^2+^ complexation by L, as the formation of ZnL^2+^ is accompanied by an increase of the absorption bands (Figure S3). 

**Figure 7:**
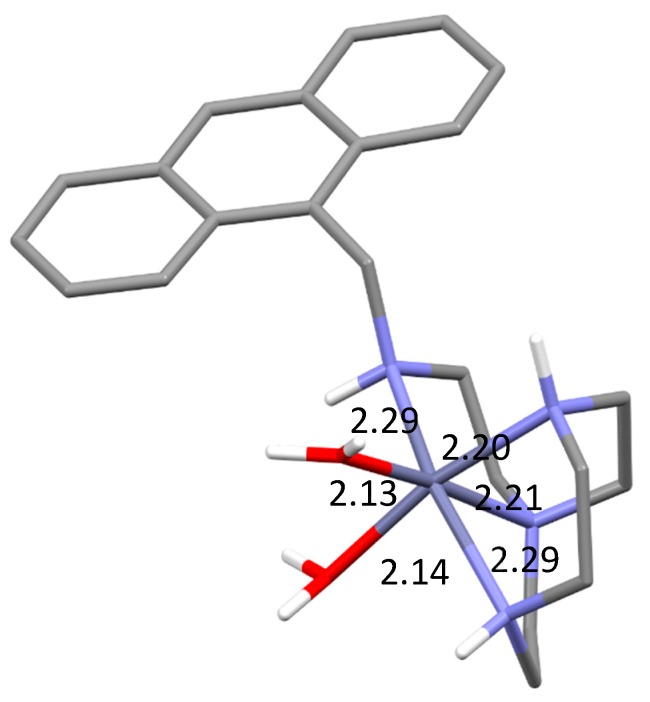
Calculated conformation for the [ZnL(H_2_O)_2_]^2+^ complex; bond distances in Å.

Modelling calculations were performed on solvated forms of ZnL^2+^ in which the coordination environment of the metal ion is completed by water molecules. The lowest energy conformer ([ZnL(H_2_O)_2_]^2+^) that was found is characterized by a distorted octahedral coordination spheres constituted by the four nitrogen atoms of the ligand and two water molecules in *cis* positions (Figure 7). The involvement of all ligand amine groups in metal coordination is in agreement with the emissive properties of the complex. The presence of two water molecules in *cis* positions should favour the coordination of chelating substrate like phosphate. On the other hand, these *cis* positions are vicinal to the anthracene group of the ligand tail, so that we can expect that binding of aromatic substrates, such as benzoate, can be favoured by stacking interactions with this group.

### 2.3. Anion Binding and Interference in Chemosensor Properties

The efficiency of a chemosensor can be affected by the interference of other species in solution. In the case of metal ions sensing, the first potential source of interference are the anions that inevitably accompany cations. To address this issue, the ability of L to act as fluorescent chemosensor for Zn^2+^ was studied in the presence of coordinating anions such as phosphate (HPO_4_^2−^, H_2_PO_4_^−^), benzoate (Bz^−^), cyanide (CN^−^) and sulphide (S^2−^).

First of all, we considered the possibility that L (in particular its protonated forms) and ZnL^2+^ interact with the anions: anions binding by both polyammonium cations and metal ion complexes is a well-known topic [55,56]. The formation of anion complexes was followed by means of potentiometric (pH-metric) titrations in 0.1 M NaCl at 298.1 ± 0.1 K. For safety reasons, the potentiometric study was limited to phosphate and benzoate. Indeed, the analysis of titration curves, performed with the program HYPERQUAD [57], showed that these anions do interact with both protonated forms of L and with its Zn^2+^ complex. Table 3 and Table 4 list, respectively, the stability constants of the anion complexes formed by the free ligand and by the complex.

Anion complexation by L (in the absence of Zn^2+^) takes place with all protonated forms of L (Table 3). The stability of the complexes increases with ligand charge, as expected for polyammonium receptors [58,59,60,61,62]. Conversely, the charge of the anions does not seem to be so important, at least for phosphate, as equal (within experimental errors) binding constants were obtained for the interaction of H_2_L^2+^ with the differently charged HPO_4_^2−^ (logK = 2.77(7)) and H_2_PO_4_^−^ (logK = 2.83(7)) anions. A similar behaviour was observed in previous studies [63,64] and was attributed to a balance between the loss of electrostatic attraction and the gain in hydrogen bonding that, in our case, is expected to occur for H_2_L(H_2_PO_4_)^+^ relative to H_2_L(HPO_4_).

Remarkably, benzoate, despite its lower charge, forms more stable complexes than phosphate with the various protonated forms of L. Such behaviour could be connected with the formation of π-stacking interactions with the anthracene residue of the ligand and/or with the hydrophobic effect deriving from the interaction of these aromatic moieties. Indeed, benzoate was recently found to form synergistic salt-bridge and π-stacking interactions that stabilize the complexes formed with polyammonium ligands containing *s*-tetrazine rings [65,66]. Anthracene offers a large surface for stacking interactions and, as a matter of fact, even neutral benzoic acid is able to form a complex of significant stability with L (log*K* = 3.4 for H_4_L^4+^ + HBz = H_3_L(HBz)^4+^, Table 3), although the loss of charge (relative to benzoate) and of the correlated electrostatic attraction gives rise to a complex stability loss of three order of magnitude (log*K* = 6.41 for H_4_L^4+^ + Bz^−^ = H_4_LBz^3+^, Table 3).

The study of solution equilibria, whose results are reported in Table 4, showed that both phosphate and benzoate are also able to interact with the complexes ZnL^2+^ and ZnHL^3+^ and, limited to benzoate, with ZnL(OH)^+^. The equilibrium constants for binding of these anions to the above receptors (ZnL^2+^, ZnHL^3+^, ZnL(OH)^+^) are invariably related to the charge of the interacting species, increasing as the charge increases on both anions and metal complexes. That is, for these systems, we observe the prevalence of coordinative over supramolecular forces. Nevertheless, in the case of benzoate, in accordance with expectations based on the calculated structures of the ZnL^2+^ complex (see above), π-stacking interactions between the aromatic moieties of the interacting species seem to make a favourable contribution, as the interaction of benzoate anion with ZnL^2+^ and ZnHL^3+^ (log*K* = 3.81 and 4.09, respectively, Table 4) is stronger than the interaction of H_2_PO_4_^−^ with the same complexes (log*K* = 3.12 and 3.38, respectively, Table 4) and even stronger than that of the more charged HPO_4_^2−^ with ZnL^2+^ (log*K* = 3.50, Table 4).

**Figure 8:**
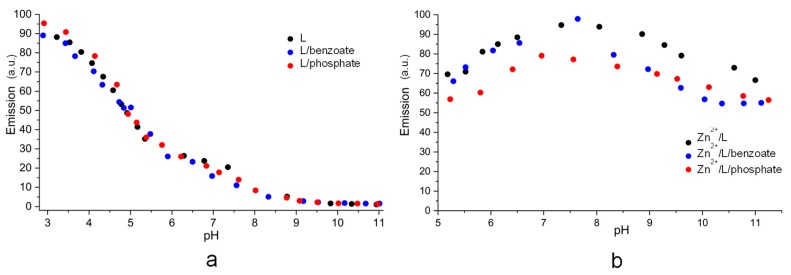
pH dependence of the emission intensity at 416 nm (λ_exc_ 362 nm) of the systems: (**a**) L, L/benzoate, L/phosphate; (**b**) Zn^2+^/L, Zn^2+^/L/benzoate, Zn^2+^/L/phosphate. [L] = [Zn^2+^] = 1 × 10^−5^ M, [benzoate] = [phosphate] = 3 × 10^−5^ M.

The fluorescence emission spectra recorded at different pH’s for the systems L/phosphate (Figure S4), L/benzoate (Figure S5), L/Zn^2+^/phosphate (Figure S6) and L/Zn^2+^/benzoate (Figure S7) show that the presence of these anions does not modify significantly the emission properties of L in the absence of Zn^2+^, while modest variations are found in the presence of the metal ion (Figure 8). In particular, phosphate gives rise to a general attenuation of the emission intensity in the pH region (6–10) useful for sensing Zn^2+^. A similar effect is produced by benzoate above pH 8, whereas no emission differences are observed at lower pH (6–7.5). Then, the chemosensor properties of L toward Zn^2+^ ions are not compromised by the presence of benzoate and phosphate anions, even when the anions are in excess (Figure 8).

**Figure 9:**
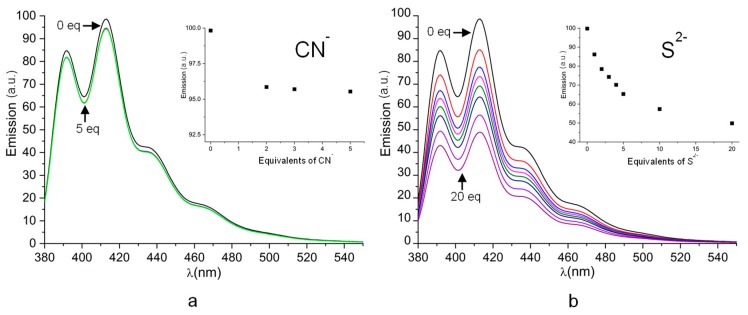
(**a**) Variation of the emission spectra of Zn^2+^/L at pH 9 upon addition of cyanide (**a**) and sulphide (**b**). [L] = [Zn^2+^] = 1 × 10^−5^ M. λ_exc_ 362 nm. Insets: Emission intensity at 416 nm versus the added equivalents of cyanide (**a**) and sulphide (**b**).

We further considered the interaction of ZnL^2+^ with CN^−^ and S^2−^ anions which are known to interact stronger than phosphate and benzoate with Zn^2+^. Since cyanide and sulphide produce extremely toxic gases (HCN, H_2_S) in acidic solutions, their effect on the emission properties of ZnL^2+^ was studied only by spectrofluorimetric titrations at pH 9. Cyanide affects very poorly the emission properties of ZnL^2+^ while sulphide produces a moderate quenching of the emission that increases with the addition of the first 5 equivalents of anion to keep almost constant upon addition of further 15 equivalents, which lead to an overall quenching of about 50% relative to the emission intensity of the original complex solution (Figure 9).

## 3. Materials and Methods

### 3.1. General

All starting materials were high purity compounds purchased from commercial sources (Merck, Milan, Italy and TCI Europe, Zwijndrecht, Belgium) and were used as supplied. 1-(2-aminoethyl)-1,4,7-triazacyclononane used for the synthesis of ligand L was prepared according to a reported procedure [67]. The ^1^H NMR spectra (400 MHz) in D_2_O solution were recorded at 25 °C on a Bruker AV400 spectrometer (Bruker, Milan, Italy). Absorption spectra were recorded at 298 K on a Jasco V-670 spectrophotometer (Jasco, Lecco, Italy). Both ligand and Zn^2+^ complex solutions were 1.0 × 10^−4^ M. Emission spectra were recorded at 298 K on a LS50B spectrofluorimeter (Perkin-Elmer, Milan, Italy). Both ligand and Zn^2+^ complex solutions were 1.0 × 10^−5^ M.

### 3.2. Synthesis of L

Anthracene-9-carbaldehyde (0.41 g, 2 mmol) was added in small portions in 1 h to a stirred solution of 1-(2-aminoethyl)-1,4,7-triazacyclononane (0.34 g, 2 mmol) in 100 cm^3^ of a 1:1 *v:v* EtOH/CH_3_CN mixture, at room temperature. The resulting solution was then kept at room temperature for 60 h before the solvent was evaporated under reduced pressure at 50 °C to leave a yellow/orange oil.

This product was dissolved in 35 cm^3^ of EtOH, heated to 50 °C and treated with 0.71 g (19 mmol) of NaBH_4_, in small portions, over 30 min with stirring. Heating (50 °C) and stirring were maintained for additional 4 h, after which, the solvent was evaporated under reduced pressure at 50 °C to leave L as an orange oil.

The whole batch of the amine was converted into the hydrochloride salt H_3_LCl_3_∙H_2_O by addition of 37% HCl solution to L dissolved in 50 cm^3^ of EtOH under stirring at room temperature. The resulting suspension was stored at 4 °C for 24 h and then separated by filtration. The solid was washed with 10 cm^3^ of Et_2_O and dried under reduced pressure at 25 °C for 12 h. Yield: 0.67 g (71%). ^1^H NMR (D_2_O, pH 2, 25 °C, 400 MHz) *δ* 2.97 (t, 4H; *J* = 5.49 Hz), 3.04 (t, 2H; *J* = 7.6 Hz), 3.23 (br 4H), 3.50 (t, 2H; *J* = 7.70 Hz), 3.54 (s, 4H), 5.46 (s, 2H), 7.70 (t, 2H; *J* = 7.55 Hz), 7.81 (t, 2H; *J* = 7.71 Hz), 8.27 (d, 2H; *J* = 8.29 Hz), 8.40 (d, 2H; *J* = 8.97 Hz) 8.85 (s, 1H). ^13^C NMR (D_2_O, pH 2, 25 °C, 400 MHz) *δ* 42.1, 42.7, 42.9, 43.7, 47.3, 49.9, 120.0, 122.5, 125.5, 127.8, 129.5, 130.1, 130.5, 130.6. Elemental analysis: Anal. Calcd. for C_23_H_29_N_4_Cl_3_O: C, 56.50%; H, 7.21%; N, 11.45%. Found: C, 56.25; H, 6.85; N, 11.39.

### 3.3. Potentiometric Measurements

Potentiometric (pH-metric) titrations, employed to determine equilibrium constants, were performed in 0.1 M NaCl at 298.1 ± 0.1 K using an automated apparatus and a procedure previously described [68,69]. The acquisition of the emf data was performed with the computer program PASAT [70]. This program is in charge of titrant additions into the measuring cell, of emf readings and of data recording. After each addition (volume set by the operator), the system allows a time interval (set by the operator) to pass before acquiring the first emf reading. Subsequent emf readings are performed at a time interval set again by the operator. When the standard deviation (σ) on the mean value of the last 10 readings and the difference between the first and the tenth readings (drift) are smaller than accepted limits (set by the operator, usually 0.05 for both parameters), the mean emf value is recorded and a new aliquot of titrant is added. If σ and/or drift exceed the selection criteria, additional emf readings are acquired until these criteria are met before reaching a maximum number of readings (set by the operator). In the event that these criteria are not met within the maximum number of readings, the system records the mean value of the last 10 readings (labelling it as a point acquired in non-equilibrium conditions) and performs a successive titrant addition. The combined Metrohm 6.0262.100 electrode was calibrated as a hydrogen-ion concentration probe by titration of previously standardized amounts of HCl with CO_2_-free NaOH solutions and determining the equivalent point by Gran’s method [71], implemented in PASAT, to obtain the standard potential, E°, and the ionic product of water (p*K*_w_ = 13.77(1) in 0.1 M NaCl at 298.1 K). The computer program HYPERQUAD [57] was used to calculate the stability constants from the potentiometric data. The concentration of L and Zn^2+^ was in the range 0.5−1 mM in all experiments, the concentration of phosphate was in the range 2–3 mM and the concentration of benzoate was in the range 1–2 mM. The studied pH range was 2.5–11.0. At least three measurements were performed for each system: firstly, the different titration curves were treated as separated curves without significant variations in the values of the calculated equilibrium constants; finally, all titration curves for each system were merged together and treated simultaneously to give the final stability constants. Different equilibrium models for the studied systems were generated by eliminating and introducing different species. Only those models for which the HYPERQUAD program furnished a variance of the residuals σ^2^ < 9 were considered acceptable. Such condition was unambiguously met by a single model for each system. Phosphate and benzoate protonation constants used in calculation where previously determined [65,72]. Ligand and anion protonation constants were necessary for the analysis of all the other equilibria involving, respectively, the ligand and the anions. Equilibrium constants for the formation of both L/anion and L/Zn^2+^ complexes were necessary for the analysis of equilibria in the L/anion/Zn^2+^ systems. Titration experiments, and relative calculations, were performed according to this order of precedence. The determined equilibrium constants are collected in Table 1, Table 2, Table 3 and Table 4, while distribution diagrams of the species formed in solution as a function of pH are shown in Figures S8–S13.

### 3.4. Spectroscopic Measurements

Absorption and emission spectra were recorded at 298 K by using a Jasco V-670 spectrophotometer and a LS50B spectrofluorimeter (Perkin-Elmer, Milan, Italy). The absorption measurements were performed with 1.0 × 10^−4^ M solutions of ligand and Zn^2+^ complex, while, for the emission spectra, ligand and Zn^2+^ complex solutions were 1.0 × 10^−5^ M or dilutions of the same.

### 3.5. Theoretical Calculations. Optimum Geometry

All calculations were conducted using version 2019-3 of the Schrödinger molecular modelling suite [73]. The X-ray crystal structures of (1-(2-Aminoethyl)-1,4,7-triazacyclononane-N,N′,N″,N‴)-(nitrato-*O*)-zinc(ii) nitrate (CSD code: BIBGOJ) [67] and cis-Diaqua-(1,4,7,10-tetra-azabicyclo(5.5.2)tetradecane)-zinc(ii) diperchlorate (CDS code: GAJFIH) [74] were used, properly modified, as input for building five- and exa-coordinated zinc complexes. One or two water molecules were initially used to complete the metal coordination sphere, but simulations converged toward the exa-coordinated [ZnL(H_2_O)_2_]^2+^ form, which was then used for the final QM/MM calculation. The diprotonated ligand was built by using the Schrödinger suite and then solvated in an orthorhombic box of TIP3P31 water molecules using the Desmond system builder implemented in Maestro Schrodinger suite. Then, 10 ns long MD simulations were carried out using the GPU-accelerated Desmond software in Maestro. Structures were extracted every 25 ps from the trajectory for a total of about 400 conformers that were clustered in ten groups. Both the lowest energy cluster representatives of the diprotonated L and the [ZnL(H_2_O)_2_]^2+^ adducts were submitted to QM/MM optimization in explicit water solvent using QSite module of the Schrodinger suite. The QM region contains the ligands or the adducts while water molecules comprised the MM region. The QM method used was B3LYP/6-31G* for all atoms in the QM region. The nature of each conformer as a true minimum was checked by frequency calculations.

## 4. Conclusions

A new scorpiand-like ligand (L) based on [9]aneN_3_ and bearing a coordinating tail terminated with an anthracene fluorophore has been prepared by a simple synthetic procedure and studied as fluorescent chemosensor for Zn^2+^. L was designed to have a rigid binding unit, the macrocyclic triamine [9]aneN_3_ which ensures the binding of Zn^2+^ without saturating its coordination environment, and a mobile lateral tail containing the sensor system. The latter consists of a fluorophore (anthracene) in close proximity to a coordinating amine group. The fluorophore is switched off when the lone pair of the close amine nitrogen is not involved in protonation or metal ion coordination, then is able to quench the excited fluorophore via a photoinduced electron transfer (PET). Accordingly, on and off switching of L was expected to occur, respectively, upon binding and release of Zn^2+^. Indeed, this strategy proved to be successful since L is able to sense Zn^2+^ in the pH range 6–10 down to nM concentrations of the metal ion.

L can efficiently sense Zn^2+^ even in the presence of large excess of coordinating anions, such as cyanide, sulphide, phosphate and benzoate, despite their ability to bind the metal ion. In ZnL^2+^, the ligand occupies only four coordination sites out of six, the remaining two positions being available for solvent molecules in the absence of stronger ligands. Anions can easily bind to these positions, but they are not able to furnish much competition with the strong L ligand. Moreover, upon coordination of the first anion, the charge of the metal ion experiences a significant neutralization and the propensity of the latter to attract further anions drops down. Nevertheless, the neutralization of charge occurring upon coordination of anions can influence in part, but not to a large extent, the strength of the metal-ligand interaction with possible, but modest, consequences on the emission properties. As a matter of fact, the bicharged S^2−^ is the anion affecting most the fluorescence emission of ZnL^2+^, but not enough to compromise the chemosensor properties of L.

Future studies will be carried out to assess the fluorescence chemosensor properties of L toward metal ions other than Zn^2+^ and the interferences that could be generated by their simultaneous presence in solution.

## Figures and Tables

**Table 1:** Protonation constants of L determined in 0.1 M NaCl aqueous solution at 298.1 ± 0.1 K. Values in parentheses are standard deviations on the last significant figures.

Equilibria	Log *K*
L + H^+^ = HL^+^	10.26 (3)
HL^+^ + H^+^ = H_2_L^2+^	7.45 (2)
H_2_L^2+^ + H^+^ = H_3_L^3+^	5.14 (2)
H_3_L^3+^ + H^+^ = H_4_L^4+^	1.74 (1)

**Table 2:** Stability constants of Zn^2+^ complexes with L determined in 0.1 M NaCl aqueous solution at 298.1 ± 0.1 K. Values in parentheses are standard deviations on the last significant figures.

Equilibria	Log *K*
L + Zn^2+^ = ZnL^2+^	11.695(8)
ZnL^2+^ + H^+^ = ZnHL^3+^	5.86 (3)
ZnL^2+^+ OH^−^ = ZnL(OH)^+^	3.95 2)
ZnL^2+^ +2OH^−^ = ZnL(OH)_2_	6.36 (2)

**Table 3:** Stability constants of anion complexes formed by L with differently protonated species of PO_4_^3−^ and with benzoate (Bz^−^) determined in 0.1 M NaCl aqueous solution at 298.1 ± 0.1 K. Values in parentheses are standard deviations on the last significant figures.

Equilibria	Log *K*
HL^+^ + HPO_4_^2−^ = HL(HPO_4_)^−^	2.23(6)
H_2_L^2+^ + HPO_4_^2−^ = H_2_L(HPO_4_)	2.77(7)
H_2_L^2+^ + H_2_PO_4_^−^ = H_2_L(H_2_PO_4_)^+^	2.83(7)
H_3_L^3+^ + H_2_PO_4_^−^ = H_3_L(H_2_PO_4_)^2+^	3.21(6)
HL^+^ + Bz^−^ = HLBz	3.05(3)
H_2_L^2+^ + Bz^−^ = H_2_LBz^+^	3.74(5)
H_3_L^3+^ + Bz^−^ = H_3_LBz^2+^	4.22(7)
H_4_L^4+^ + Bz^−^ = H_4_LBz^3+^	6.41(8)
H_4_L^4+^ + HBz = H_4_L(HBz)^4+^	3.4(1)

**Table 4:** Stability constants of anion complexes formed by ZnL^2+^ with differently protonated species of PO_4_^3−^ and with benzoate (Bz^−^) determined in 0.1 M NaCl aqueous solution at 298.1 ± 0.1 K. Values in parentheses are standard deviations on the last significant figures.

Equilibria	Log *K*
ZnL^2+^ + PO_4_^3−^ = ZnL(PO_4_) ^−^	5.12(6)
ZnL^2+^ + HPO_4_^2−^ = ZnL(HPO_4_)	3.50(7)
ZnL^2+^ + H_2_PO_4_^−^ = ZnL(H_2_PO_4_)^+^	3.12(7)
ZnHL^3+^ + H_2_PO_4_^−^ = ZnHL(H_2_PO_4_)^2+^	3.38(6)
ZnL^2+^ + Bz^−^ = ZnLBz^+^	3.81(1)
ZnHL^3+^ + Bz^−^ = ZnHLBz^2+^	4.09(3)
ZnL(OH)^+^ + Bz^−^ = ZnL(OH)Bz	3.49(3)

## Supplementary Materials

The following are available online: distribution diagrams of the species formed in the systems L/H^+^, L/Zn^2+^/H^+^, L/phosphate/H^+^, L/benzoate/H^+^, L/Zn^2+^/phosphate/H^+^ and L/Zn^2+^/benzoate/H^+^, absorption spectra of L and Zn^2+^/L, emission spectra of L/phosphate, L/benzoate, L/Zn^2+^/phosphate and L/Zn^2+^/benzoate, data for the determination of the limit of detection of Zn^2+^.
