# Supplementary material for: Sensing Zn2+ in Aqueous Solution with a Fluorescent Scorpiand Macrocyclic Ligand Decorated with an Anthracene Bearing Tail
Source: Molecules. 2020 Mar 17;25(6):1355. doi: 10.3390/molecules25061355 (PMC7146481; doi:10.3390/molecules25061355)
Supplement: Supplementary file 1 [file molecules-25-01355-s001.pdf]

# Supplementary Materials

# Sensing $Zn^{2+}$ in aqueous solution with a fluorescent scorpiand macrocyclic ligand decorated with an anthracene bearing tail.

**Matteo Savastano[1], Matteo Fiaschi[1], Giovanni Ferraro,[1] Paola Gratteri[2], Palma Mariani[1], Antonio Bianchi[1,*], Carla Bazzicalupi[1]**

[1] Department of Chemistry "Ugo Schiff", University of Florence, Via della Lastruccia, 3-13, 50019 Sesto Fiorentino (Italy).

[2] Department of NEUROFARBA- Pharmaceutical and Nutraceutical section, and Laboratory of Molecular Modeling Cheminformatics & QSAR, University of Florence, Via Ugo Schiff 6, 50019 Sesto Fiorentino (Italy).

# Content

Figure S1. Absorption spectra of L at different pH values. …………………………………… S3
Figure S2. Plot of the fluorescence intensity at 413 nm versus the concentration of $Zn^{2+}$ and data fitting for the determination of the limit of detection (LOD). …………………………… S4
Figure S3. Absorption spectra of the $Zn^{2+}$/L system at different pH values. …………………. S5
Figure S4. Emission spectra of the system L/phosphate at different pH values. …………….. S6
Figure S5. Emission spectra of the system L/benzoate at different pH values. …………..…... S7
Figure S6. Emission spectra of the system L/$Zn^{2+}$/phosphate at different pH values. …..…... S8
Figure S7. Emission spectra of the system L/$Zn^{2+}$/benzoate at different pH values. ……...…. S9
Figure S8. Distribution diagram of the species formed by L as a function of pH. ………….. S10
Figure S9. Distribution diagram of the species formed by L with $Zn^{2+}$ as a function of pH. .. S11
Figure S10. Distribution diagram of the species formed by L with phosphate as a function of pH. ………………………………………………………………………………………… S12
Figure S11. Distribution diagram of the species formed by L with benzoate as a function of pH. ………………………………………………………………………………………… S13
Figure S12. Distribution diagram of the species formed by L with $Zn^{2+}$ and phosphate as a function of pH. ………………………………………………………………………………… S14
Figure S13. Distribution diagram of the species formed by L with $Zn^{2+}$ and benzoate as a function of pH. ………………………………………………………………………………… S15

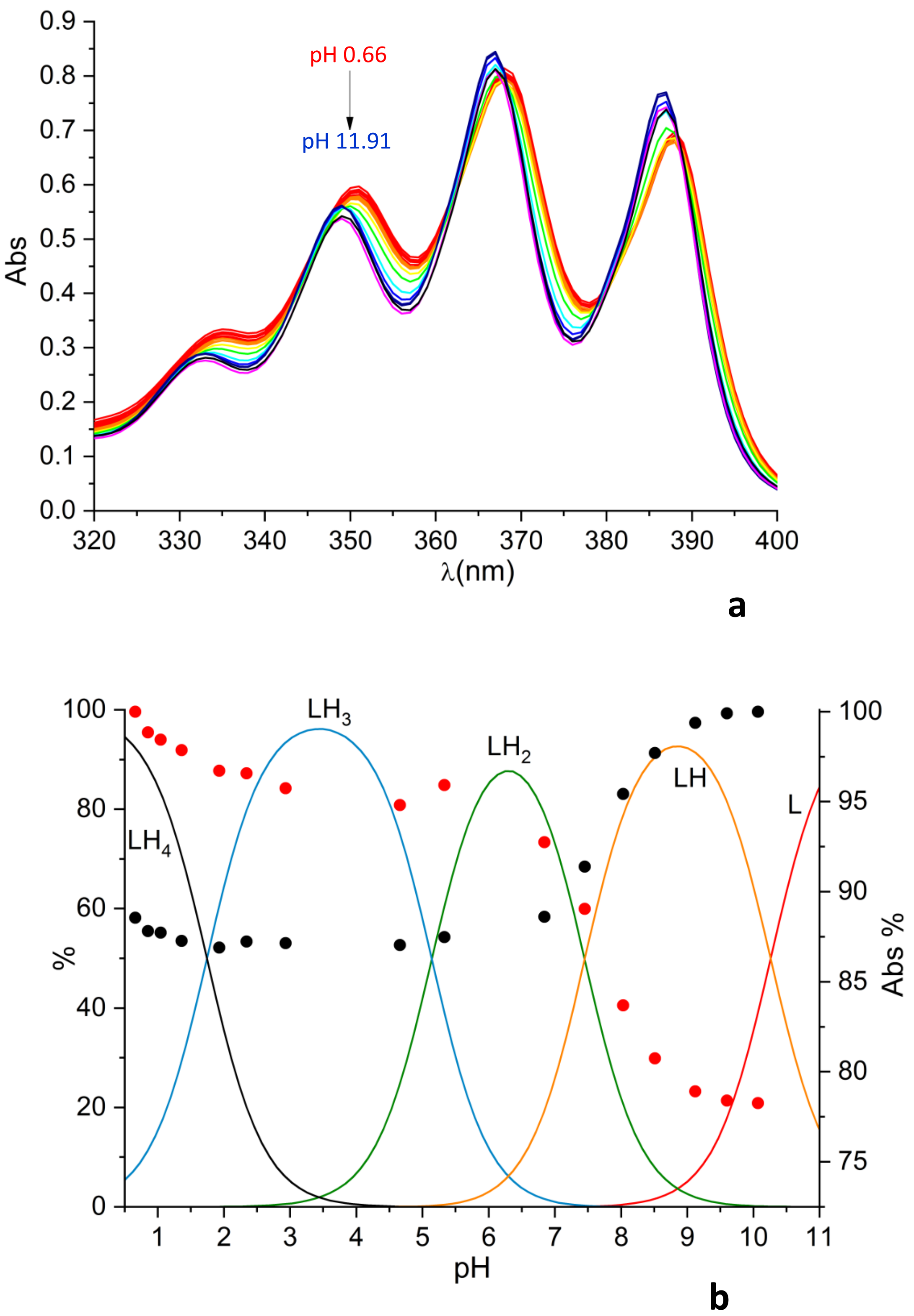


Figure S1. a) Absorption spectra of L at different pH values. [L] = 1×10$^{-4}$ M. b) Absorbances at 387 nm (black dots) and 356 nm (red dots) superimposed to the distribution diagram of the species formed by L as a function of pH. [L] = 1×10$^{-4}$ M).

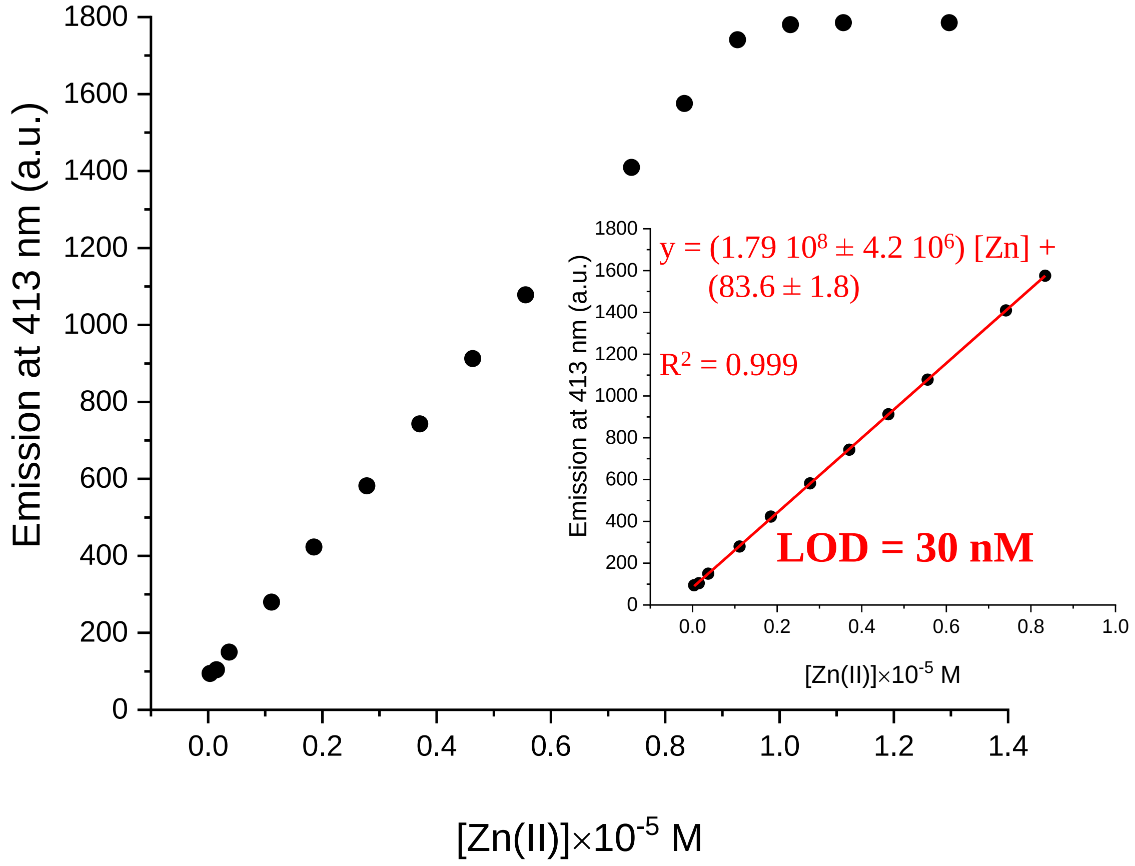


Figure S2. Plot of the fluorescence intensity at 413 nm ($\lambda_{exc}$ 362 nm) of L ($1\times10^{-5}$M, pH 9) versus the concentration of $Zn^{2+}$. Inset: data fitting for the determination of the limit of detection (LOD) based on the 3σ/slope.

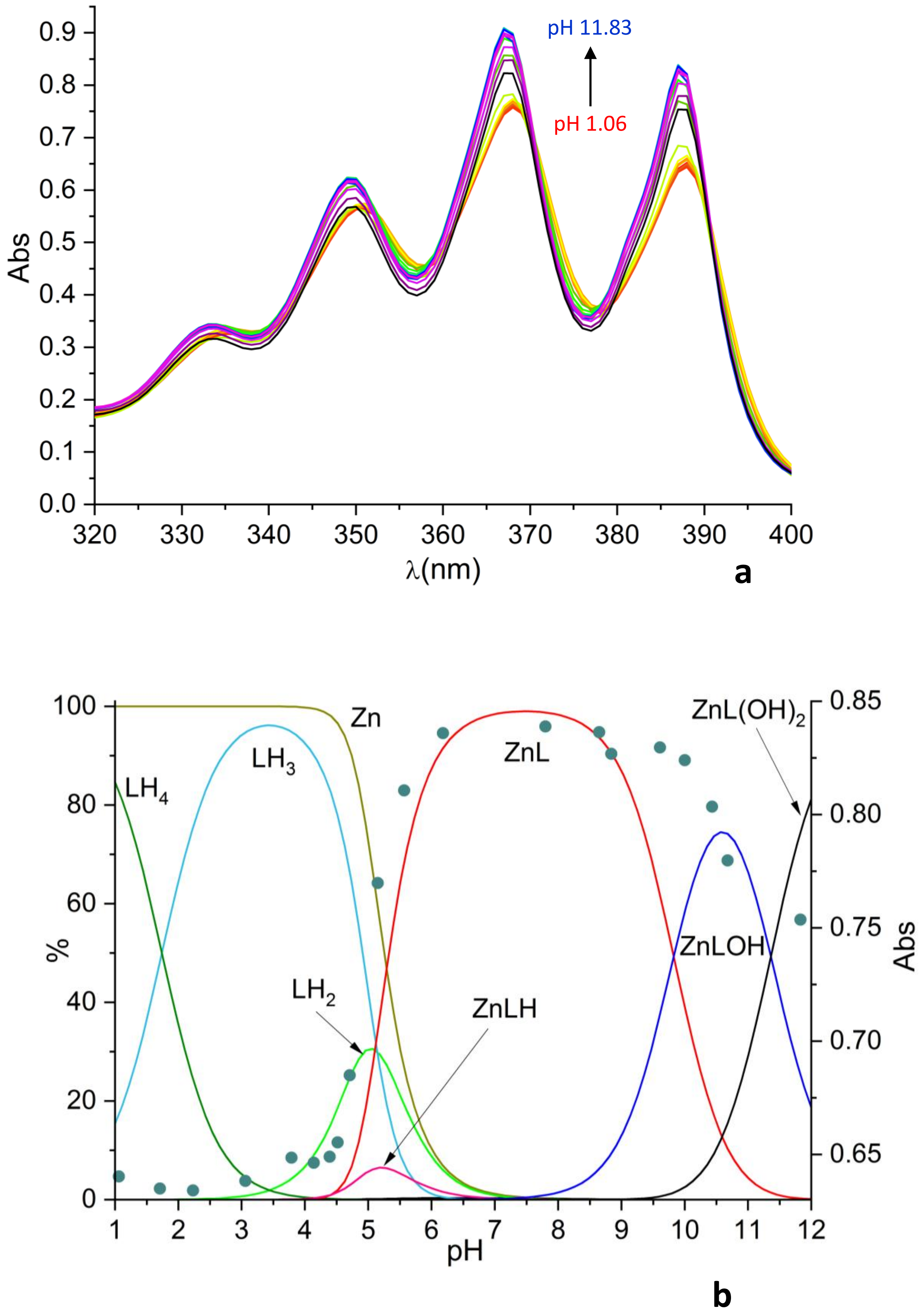


Figure S3. a) Absorption spectra of the $Zn^{2+}$/L system at different pH values. [L] = [$Zn^{2+}$] = $1\times10^{-4}$ M. b) Absorbance at 367 nm (green dots) superimposed to the distribution diagram of the species formed in the $Zn^{2+}$/L system ([L] = [$Zn^{2+}$] = $1\times10^{-4}$ M).

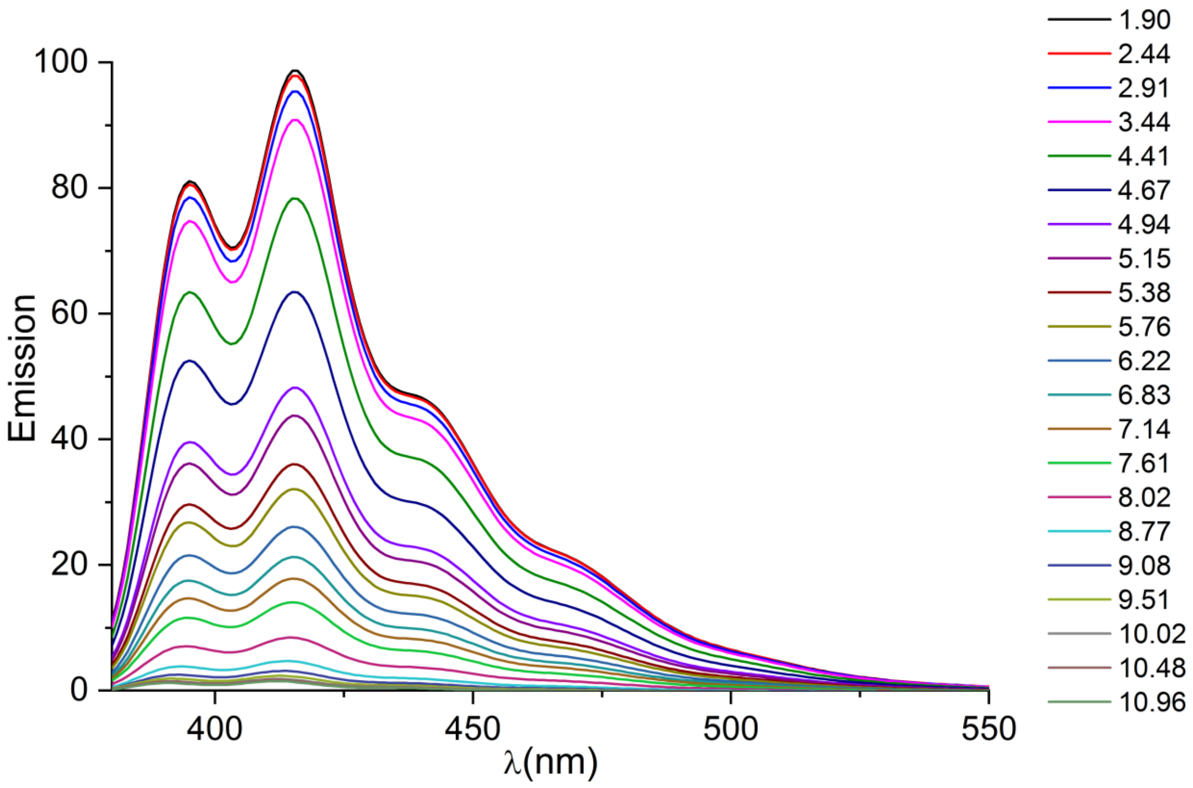


Figure S4. Emission spectra of the system L/phosphate at different pH values. [L] = [phosphate] = $1\times10^{-5}$ M. $\lambda_{exc}$ 362 nm.

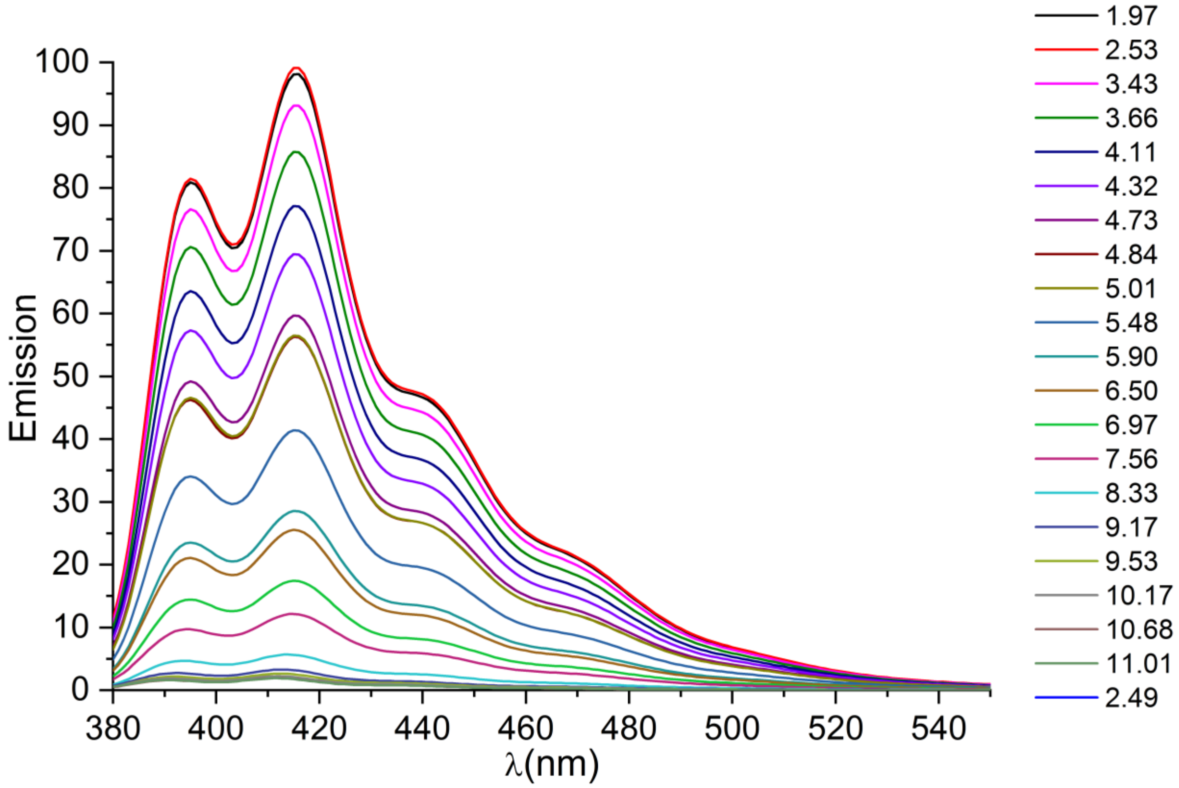


Figure S5. Emission spectra of the system L/benzoate at different pH values. [L] = [benzoate] = $1{\times}10^{-5}$ M. $\lambda_{exc}$ 362 nm.

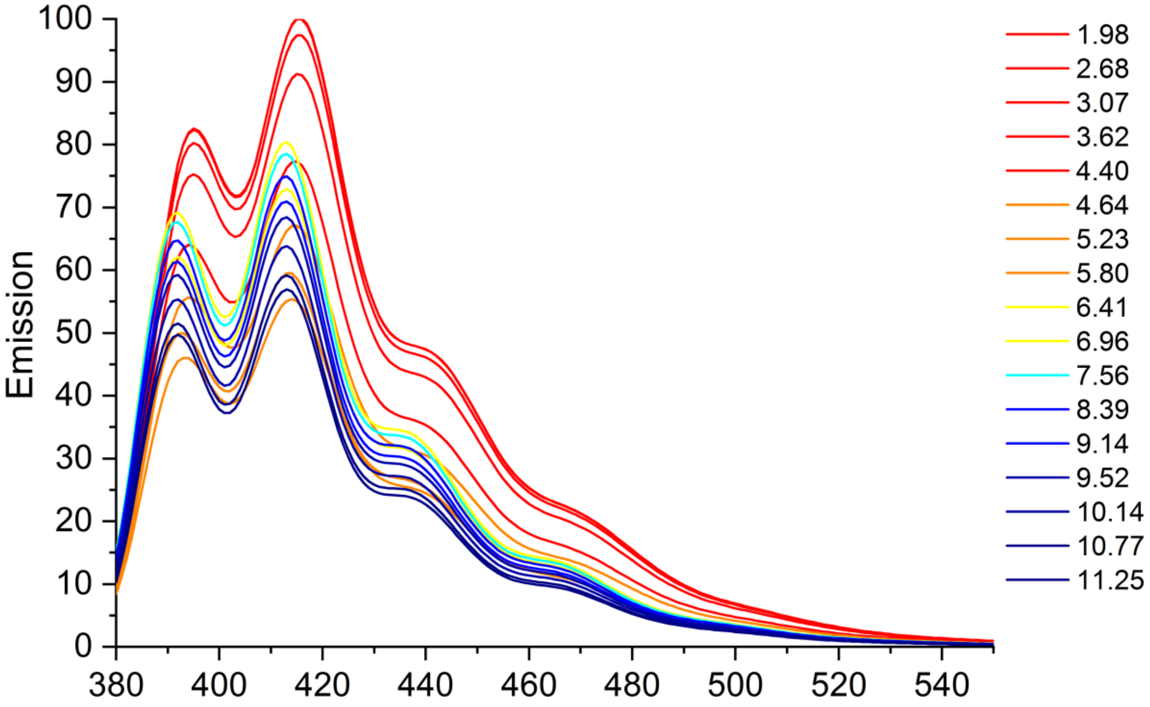


Figure S6. Emission spectra of the system L/$Zn^{2+}$/phosphate at different pH values. [L] = [$Zn^{2+}$] = [phosphate] = $1\times10^{-5}$ M. $\lambda_{exc}$ 362 nm.

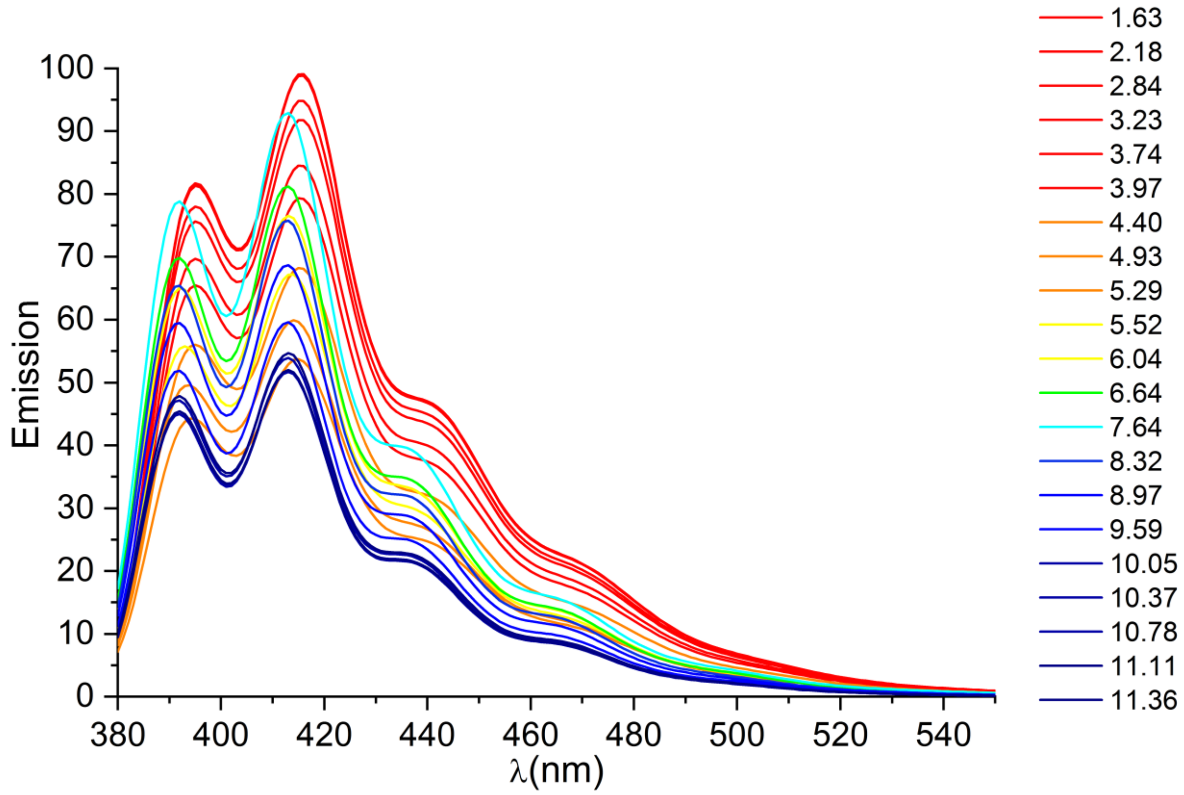


Figure S7. Emission spectra of the system L/$Zn^{2+}$/benzoate at different pH values. [L] = [$Zn^{2+}$] = [benzoate] = $1\times10^{-5}$ M. $\lambda_{exc}$ 362 nm.

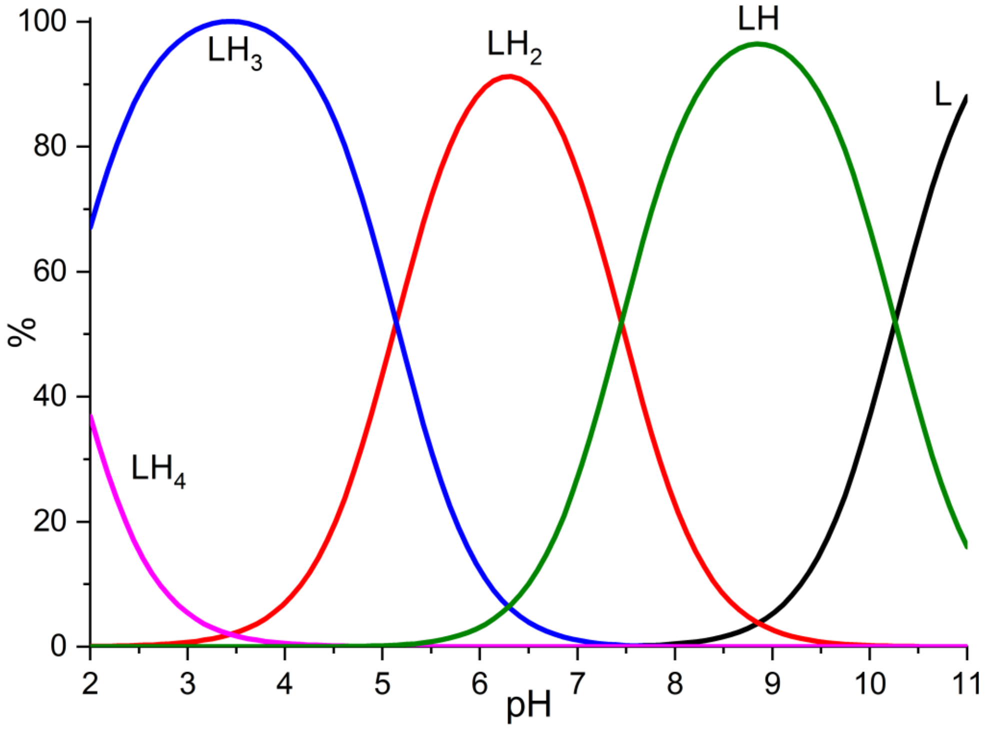


Figure S8. Distribution diagram of the species formed by L as a function of pH. [L] = 1×10$^{-3}$ M. Charges omitted.

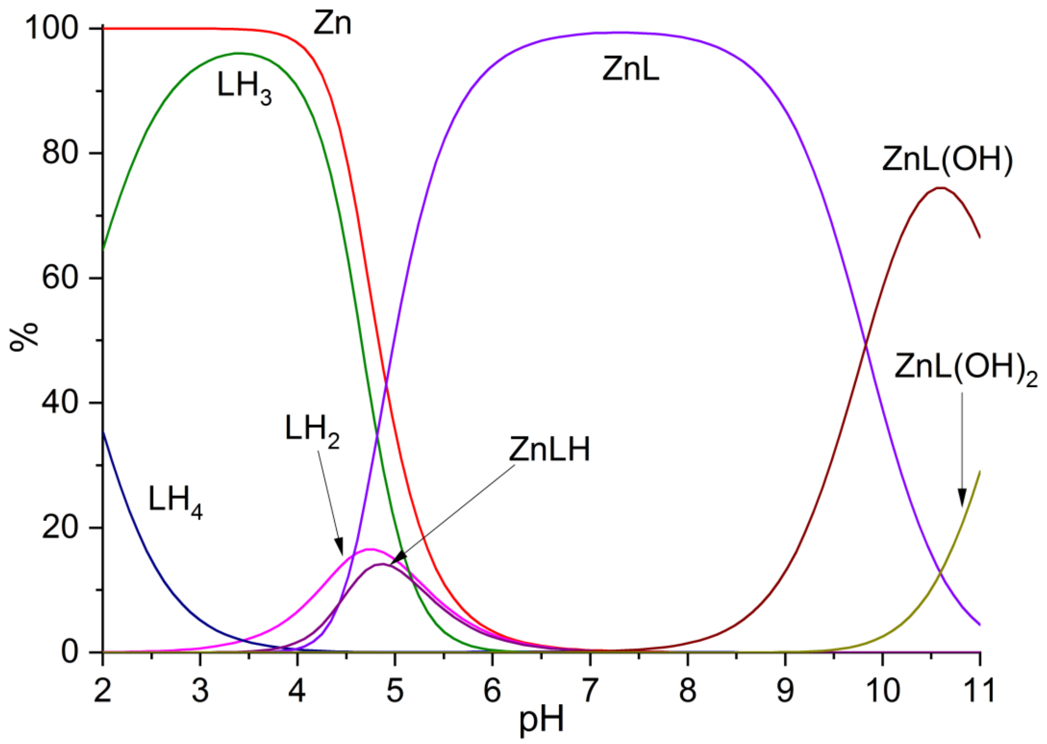


Figure S9. Distribution diagram of the species formed by L with $Zn^{2+}$ as a function of pH. [L] = [$Zn^{2+}$] = $1\times10^{-3}$ M. Charges omitted.

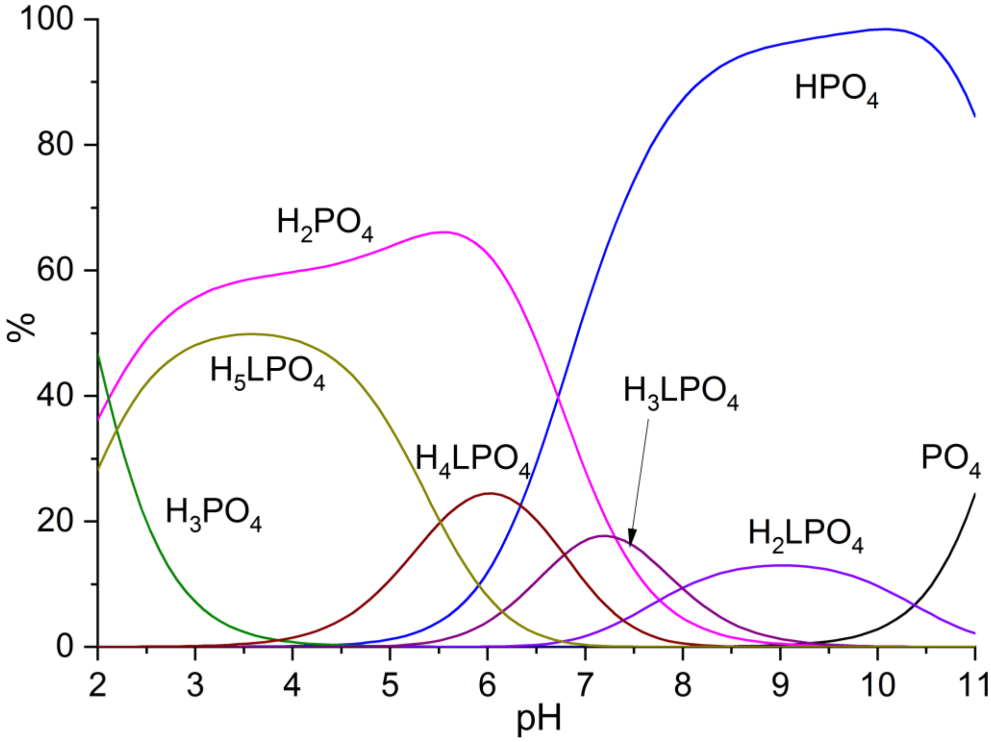


Figure S10. Distribution diagram of the species formed by L with phosphate as a function of pH. [L] = [phosphate] = $1\times10^{-3}$ M. Charges omitted.

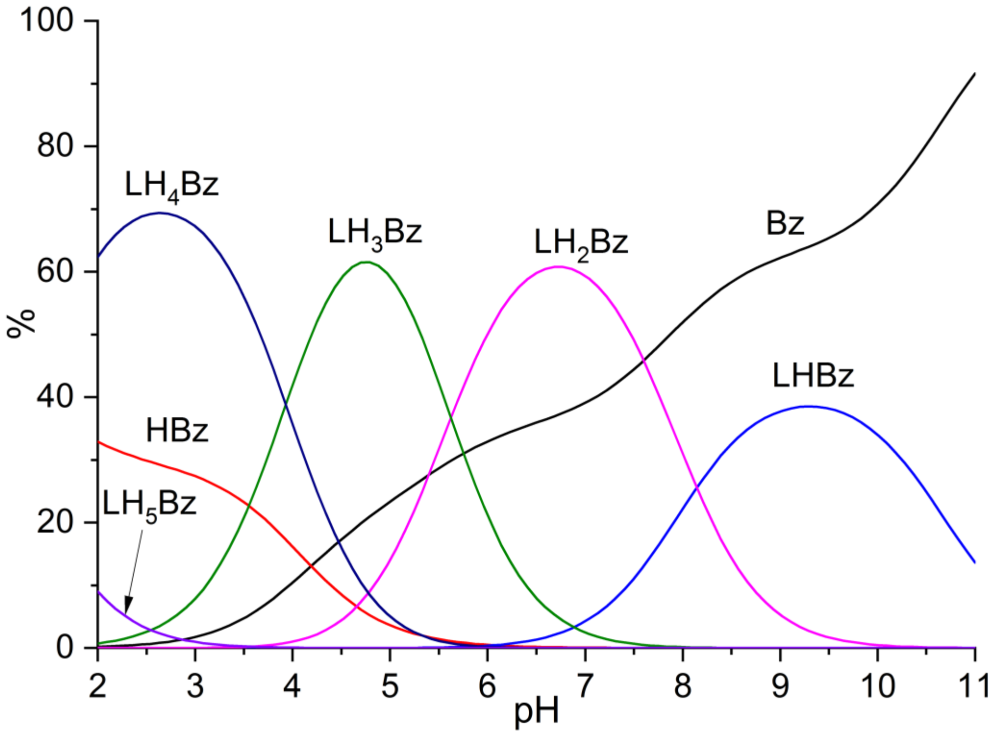


Figure S11. Distribution diagram of the species formed by L with benzoate as a function of pH. [L] = [benzoate] = $1\times10^{-3}$ M. Charges omitted.

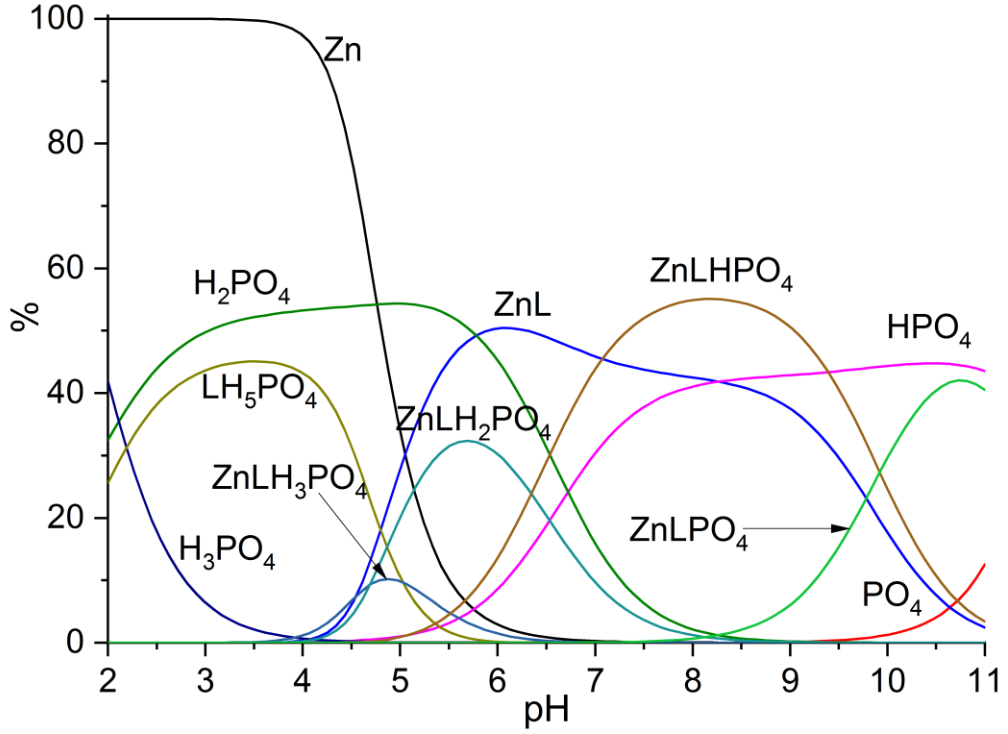


Figure S12. Distribution diagram of the species formed by L with $Zn^{2+}$ and phosphate as a function of pH. [L] = [$Zn^{2+}$] = [phosphate] = $1\times10^{-3}$ M. Charges omitted.

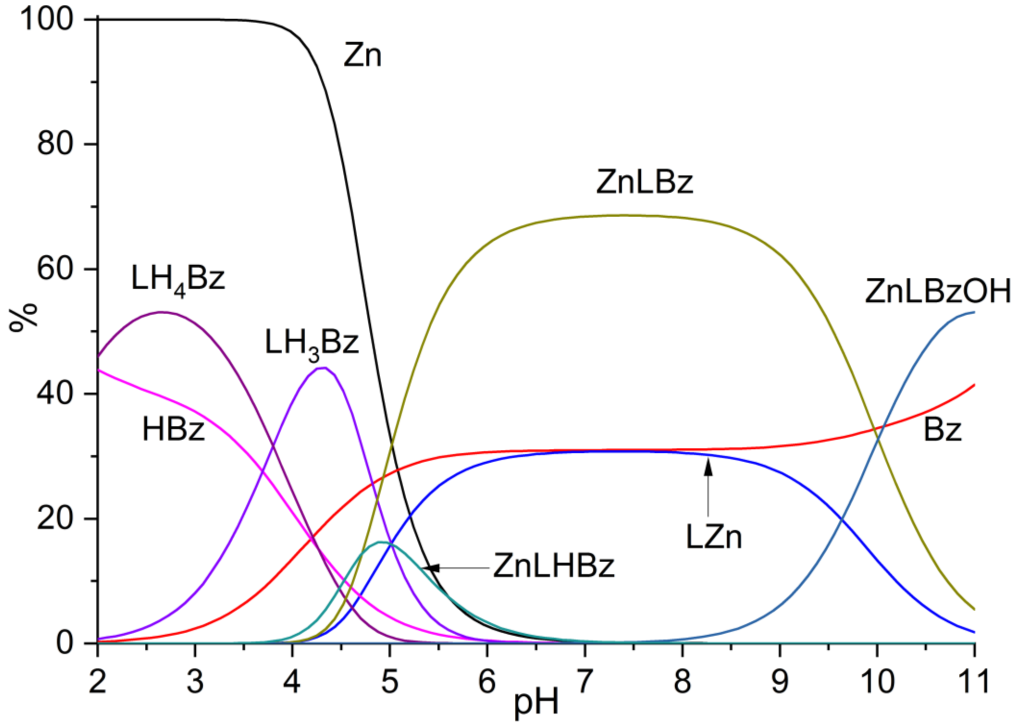


Figure S13. Distribution diagram of the species formed by L with $Zn^{2+}$ and benzoate as a function of pH. [L] = [$Zn^{2+}$] = [benzoate] = $1\times10^{-3}$ M. Charges omitted.
